# Ephedrae Herba as a potential source of SARS-CoV-2 RNA-dependent RNA polymerase inhibitory activity

**DOI:** 10.3389/fphar.2026.1718569

**Published:** 2026-07-02

**Authors:** Yuka Kiba, Tsuyoshi Hayashi, Hirokazu Ando, Hitoshi Kamauchi, Takami Yokogawa, Ryuichiro Suzuki, Takashi Tanikawa, Masashi Kitamura

**Affiliations:** 1 Faculty of Pharmacy and Pharmaceutical Sciences, Josai University, Sakado, Saitama, Japan; 2 Department of Virology II, National Institute of Infectious Diseases, Japan Institute for Health Security, Musashimurayama, Tokyo, Japan; 3 Faculty of Pharmacy, Institute of Medical, Pharmaceutical and Health Sciences, Kanazawa University, Kanazawa, Ishikawa, Japan; 4 Research Institute of Pharmaceutical Sciences, Musashino University, Nishitokyo-shi, Tokyo, Japan; 5 College of Pharmaceutical Sciences, Ritsumeikan University, Kusatsu, shiga, Japan

**Keywords:** COVID-19, Ephedrae Herba, kampo, Maoto, RNA-dependent RNA polymerase, SARS-CoV-2

## Abstract

**Introduction:**

Maoto (麻黄湯) is a traditional Japanese Kampo formula composed of four crude drugs—Ephedrae Herba, Armeniacae Semen, Cinnamomi Cortex, and Glycyrrhizae Radix—and has been used to treat febrile viral illnesses. Among these, Ephedrae Herba has been associated with antiviral activity in previous studies. However, little is known about the effects of Ephedrae Herba on viral infection, spike (S) protein-mediated syncytium formation (cell–cell fusion), and viral enzymes, or the specific metabolites underlying these activities. In this study, we focused on Ephedrae Herba and comparatively evaluated the effects of the four crude drugs of Maoto on SARS-CoV-2 infection, S protein-mediated cell–cell fusion, and virus replication-related processes.

**Methods:**

We evaluated the inhibitory effects of the four crude drugs of Maoto on SARS-CoV-2 infection in VeroE6/TMPRSS2 cells. S protein-mediated cell–cell fusion was evaluated using a split-luciferase assay in HEK293T cells. The inhibitory effects on viral enzymes, including 3C-like protease, papain-like protease, and RNA-dependent RNA polymerase (RdRp), were assessed using *in vitro* enzymatic assays.

**Results:**

Comparative analyses of the four crude drugs showed that Ephedrae Herba, Armeniacae Semen, and Cinnamomi Cortex suppressed SARS-CoV-2 infection in VeroE6/TMPRSS2 cells, whereas Glycyrrhizae Radix inhibited S protein-mediated cell–cell fusion in HEK293T cells. In the biochemical assays, Ephedrae Herba and Cinnamomi Cortex showed inhibitory activity against SARS-CoV-2 RdRp. Further analysis of Ephedrae Herba showed that its high-polarity fraction exhibited concentration-dependent inhibitory activity against both SARS-CoV-2 and norovirus RdRp with IC_50_ values of 8.6 and 16.5 μg/mL, respectively.

**Conclusion:**

Ephedrae Herba contains high-polarity metabolites associated with RdRp-related inhibitory activity in biochemical assays. Nevertheless, further studies are needed to determine the extent to which this activity contributes to the effects observed in cells.

## 1 Introduction

Kampo medicine is a traditional Japanese therapeutic system in which standardized crude drugs are combined into fixed formulas according to symptoms and disease conditions. More than 100 crude drugs are listed in the Japanese Pharmacopoeia, where their botanical origins, medicinal parts, and quality standards are defined (Japanese Pharmacopoeia 18th Edition | Pharmaceuticals and Medical Devices Agency, n.d.). Among these crude drugs, Ephedrae Herba, the dried stem of Ephedra species, is a principal crude drug in Kampo formulas for common cold- and febrile illness-related symptoms, including Maoto (麻黄湯) and Kakkonto (葛根湯).

Maoto (麻黄湯) is a representative Kampo formula composed of Ephedrae Herba, Cinnamomi Cortex (bark of *Cinnamomum cassia*), Armeniacae Semen (seeds of *Prunus armeniaca*), and Glycyrrhizae Radix (roots and stolons of *Glycyrrhiza uralensis* and related species). It has traditionally been used to treat the early symptoms of viral febrile illnesses, including influenza and the common cold, particularly fever, cough, and chills (Nishimura et al., 2009). Studies have shown that it alleviates influenza symptoms and reduces tissue damage in animal models and clinical trials (Nabeshima et al., 2012; Nishi et al., 2021). Clinical studies have demonstrated comparable therapeutic effectiveness of neuraminidase inhibitors, such as oseltamivir and zanamivir, in shortening the duration of fever and mitigating influenza symptoms (Nabeshima et al., 2012).

Maoto has also been used in patients with COVID-19 to alleviate key symptoms such as airway inflammation and sputum production. A study on post-exposure prophylaxis for COVID-19 using Maoto found that it significantly reduced the rate of infection in healthcare workers exposed to the virus (Nabeshima et al., 2022). The prophylactic efficacy of Maoto was reported to be 84.5%, with no serious adverse reactions (Nabeshima et al., 2022).

SARS-CoV-2 initiates infection by engaging the angiotensin-converting enzyme 2 (ACE2) receptor on the host cell surface through its spike (S) protein (Shang et al., 2020). This interaction promotes viral entry through membrane fusion, aided by host factors such as transmembrane protease serine 2 (TMPRSS2). In addition to mediating viral entry, the SARS-CoV-2 S protein can also drive cell–cell fusion, leading to syncytia formation, a pathological phenotype associated with disease progression (Chaudhary et al., 2023; Li H. et al., 2024). Following entry, SARS-CoV-2 replication depends on essential viral enzymes, including 3C-like protease (3CLpro), papain-like protease (PLpro), and RNA-dependent RNA polymerase (RdRp), which are required for viral polyprotein processing and RNA synthesis (Anirudhan et al., 2021; Zhu et al., 2022).

Recent studies have shown promising results regarding Maoto’s ability to suppress SARS-CoV-2 infection in cell cultures. Maoto was the most effective among the tested Kampo formulas in inhibiting SARS-CoV-2 infection in VeroE6/TMPRSS2 cells (Kakimoto et al., 2022). Ephedrae Herba played a significant role in suppressing SARS-CoV-2 infection compared with other crude drug extracts such as Scutellariae Radix, Paeoniae Radix, and Glycyrrhizae Radix (Kakimoto et al., 2022). However, the specific viral replication-related factors affected by Ephedrae Herba, as well as the metabolites underlying these effects, remain unclear.

In this study, we focused on Ephedrae Herba, for which previous studies suggest antiviral potential. We evaluated its effects in cell-based SARS-CoV-2 infection assays, an S protein-mediated cell–cell fusion assay, and *in vitro* enzymatic assays targeting 3CLpro, PLpro, and RdRp. Because each crude drug contains multiple plant metabolites, the four crude drugs of Maoto were evaluated in parallel in each assay system, allowing comparison of their inhibitory effects. We further investigated the RdRp-related inhibitory activity of Ephedrae Herba using chemically distinct *Ephedra* samples and fractionated extracts. To further characterize the polymerase-related activity of Ephedrae Herba, we also examined its effects on norovirus RdRp.

## 2 Materials and methods

### 2.1 Materials

Ephedrae Herba (Lot: GA20608), Cinnamomi Cortex (Lot: 403022), and Glycyrrhizae Radix (Lot: 402905) were purchased from Uchida Wakan-yaku Co., Ltd. (Tokyo, Japan). Armeniacae Semen (Lot: 2518001) was procured from Tochimoto Tenkaido Co., Ltd. (Osaka, Japan). These crude drugs were used as pharmacopoeia-grade materials compliant with the Japanese Pharmacopoeia. Their botanical origins were confirmed based on supplier information and pharmacopoeial standards (Japanese Pharmacopoeia 18th Edition | Pharmaceuticals and Medical Devices Agency, n.d.). The materials used in this study were Ephedrae Herba, the dried stem of *Ephedra sinica* Stapf [Ephedraceae] (voucher no. JU_EH1); Cinnamomi Cortex, the bark of *C. cassia* (L.) J. Presl [Lauraceae] (voucher no. JU_CC1); Glycyrrhizae Radix, the root and stolon of *G. uralensis* Fisch. ex DC. [Fabaceae] (voucher no. JU_GR1); and Armeniacae Semen, the seed of *P. armeniaca* L. [Rosaceae] (voucher no. JU_PA1). Voucher specimens were deposited at the Medicinal Plant Garden, Faculty of Pharmaceutical Sciences, Josai University, Japan.

*Ephedra sinica* samples (voucher nos. KES1–8), representing distinct strains with different phytochemical profiles, were cultivated and maintained at the Medicinal Plant Garden, School of Pharmacy, College of Pharmaceutical Sciences, Kanazawa University, Japan. The plants were authenticated by Dr. Hirokazu Ando, and the collected specimens were deposited in the herbarium of the same garden.

The crude drugs or dried stems of *E. sinica* (10 g) were refluxed with 300 mL of 70% EtOH for 1 h, and the resultant extracts were dried by evaporation. Samples were prepared by dissolving the extracts in dimethyl sulfoxide (DMSO) at 10 mg/mL and were stored at 4 °C until use. The crude drug extracts used for the comparative *in vitro* screening assay were prepared as previously described (Kiba et al., 2021). The original library comprised 124 crude drug extracts, of which 120 with sufficient remaining sample material were used in the present study.

### 2.2 SARS-CoV-2 infection in cell culture

Infection experiments using SARS-CoV-2 were conducted using imaging-based analysis, as described previously (Hayashi et al., 2021; Hayashi et al., 2025; Kiba et al., 2023). VeroE6/TMPRSS2 cells (JCRB 1819; JCRB Cell Bank, Osaka, Japan), which stably express TMPRSS2, were maintained in Dulbecco’s Modified Eagle Medium (DMEM) supplemented with 10% fetal bovine serum (FBS) and 1 mg/mL G418 (Nacalai Tesque, Kyoto, Japan). The SARS-CoV-2 strain used in this study, 2019-nCoV/Japan/TY/WK-521/2020 (WK-521), was propagated in VeroE6/TMPRSS2 cells, and viral titers were determined using immunofluorescence analysis. For the infection assay, VeroE6/TMPRSS2 cells were seeded into 96-well plates and used after reaching confluence. The confluent cell monolayers were pretreated with test samples for 1 h at 37 °C. The cells were then infected with SARS-CoV-2 at a multiplicity of infection (MOI) of 0.5–0.8 and incubated for 24 h at 37 °C. The final concentrations of crude drug extracts were 12.5, 25, and 50 μg/mL. After incubation, cells were fixed with 4% paraformaldehyde and permeabilized with 0.2% Triton X-100. Viral infection was detected by immunofluorescence staining using a rabbit monoclonal antibody against the receptor-binding domain of the SARS-CoV-2 S protein (1:3,000 dilution; clone HL1003, GTX635792; GeneTex, Irvine, CA, USA), followed by an Alexa Fluor 488-conjugated goat anti-rabbit IgG secondary antibody (1:1,000 dilution; Life Technologies, Carlsbad, CA, USA). Nuclei were counterstained with 4',6-diamidino-2-phenylindole (DAPI). Images were acquired using the Operetta CLS High-Content Analysis System (PerkinElmer, Waltham, MA, USA). In parallel with the evaluation of viral infectivity, cell number was evaluated by counting DAPI-positive nuclei, and the percentage of infected cells (spike-positive/DAPI-positive) was quantified using the Harmony software (PerkinElmer).

### 2.3 Evaluation of crude drug effects on SARS-CoV-2 S protein-mediated cell–cell fusion

The effect of the samples on SARS-CoV-2 S protein-mediated cell–cell fusion was evaluated based on a split-luciferase reporter system. HEK293T cells (RBRC-RCB2202, RIKEN BRC, Ibaraki, Japan) were cultured in DMEM supplemented with 10% FBS, 100 U/mL penicillin, and 100 μg/mL streptomycin at 37 °C in a humidified atmosphere containing 5% CO_2_. Cells were seeded into 24-well plates at a density of 1.0 × 10^4^ cells/well and incubated for 24 h. Donor cells were transfected with 125 ng of pcDNA3.1-SARS2-Spike vector (a gift from Fang Li; Addgene plasmid #145032; http://n2t.net/addgene:145032; RRID:Addgene_145,032) and 125 ng of pBiT3.1-N [CMV/HiBiT/Blast] vector (Promega, Madison, WA, USA) using TransIT-293 (Takara, Shiga, Japan). Acceptor cells were transfected with 125 ng of pcDNA3.1-hACE2 (a gift from Fang Li; Addgene plasmid #145033; http://n2t.net/addgene:145033; RRID:Addgene_145,033) and 125 ng of the LgBiT Expression Vector (Promega) using the same transfection reagent. At 20 h post-transfection, the culture medium of both the donor and acceptor cells was replaced. Donor cells were then mixed with acceptor cells in the presence or absence of test samples. Four hours after mixing, the Nano-Glo® Live Cell Assay System (Promega) was added to the culture medium, and luminescence based on HiBiT/LgBiT complementation was measured using a Synergy H1 microplate reader (Agilent Technologies, Wilmington, DE, USA). Cell viability was assessed using the Cell Count Reagent SF kit (Nacalai Tesque) according to the manufacturer’s instructions. Batimastat was used as a positive control (Hörnich et al., 2021) (Supplementary Figure S1).

### 2.4 Enzymatic assays for SARS-CoV-2 3CLpro and PLpro

Enzymatic assays targeting SARS-CoV-2 3CLpro and PLpro were performed based on previous reports with slight modifications (Iketani et al., 2021; Kiba et al., 2024). The gene encoding SARS-CoV-2 PLpro (amino acid residues 746–1064 of NSP3, NCBI Reference Sequence: YP_009725299) was codon-optimized for efficient expression in *Escherichia coli*, and a GST tag was fused to its N-terminus. The resulting expression plasmid (pGEX-6p-1-SARS-CoV-2-PLpro) was obtained from GenScript (Piscataway, NJ, USA) (Kiba et al., 2024). A codon-optimized expression vector encoding an N-terminal GST-tagged SARS-CoV-2 3CLpro (pGEX-5X-1-SARS-CoV-2-3CLpro) was obtained from GenScript based on a previous report (Iketani et al., 2021). The pGEX-6p-1-SARS-CoV-2-PLpro plasmid or pGEX-5X-1-SARS-CoV-2-3CLpro was transformed into *E. coli* BL21 (DE3) cells. Cultures were grown at 37 °C until the optical density at 600 nm (OD_600_) reached 0.6, followed by induction with 1 mM Isopropyl β-D-1-thiogalactopyranoside (IPTG) and incubation at 16 °C with shaking at 120 rpm for 20 h. After incubation, the cells were harvested by centrifugation at 3,000 × g for 10 min at 4 °C and washed with cold Dulbecco’s phosphate-buffered saline (D-PBS). The pellet was resuspended in lysis buffer (0.5% Triton X-100, 20 mM Tris-HCl, pH 7.5) and homogenized by sonication (3 cycles of 30 s each). The lysate was centrifuged at 15,000 × g for 10 min at 4 °C to obtain the supernatant. PLpro was purified using a GST-accept resin (Nacalai Tesque) according to the manufacturer’s instructions, followed by cleavage of the GST tag with PreScission protease (Cytiva, Tokyo, Japan). Because 3CLpro lost significant enzymatic activity during the GST removal process, GST-3CLpro was used for the enzymatic assay.

The fluorogenic substrates Ac-Thz-Tle-Leu-Gln-MCA (for 3CLpro) and Z-Arg-Leu-Arg-Gly-Gly-MCA (for PLpro; both obtained from the Peptide Institute, Osaka, Japan) were used in the enzymatic assays. Reaction mixtures consisted of the purified enzyme, assay buffer (20 mM Tris-HCl pH 7.5, 1 mM ethylenediaminetetraacetic acid (EDTA), and 150 mM NaCl for 3CLpro; 20 mM Tris-HCl pH 7.5, 10 mM dithiothreitol [DTT], and 150 mM NaCl for PLpro), and 10 µM of the appropriate fluorogenic substrate. The reactions were incubated at 37 °C for 30 min. Fluorescence was measured every 3 min at an excitation wavelength of 380 nm and emission wavelength of 460 nm using a microplate reader. The purified recombinant enzymes were confirmed by 12% SDS-PAGE and validated using the known inhibitors GRL-0617 for PLpro and ebselen for 3CLpro (Supplementary Figure S2).

### 2.5 Fluorescent RdRp activity assay

Plasmid pRSFDuet-1 (nsp8-nsp7) (nsp12) was kindly provided by Marc Delarue (Addgene Plasmid #165451) (Madru et al., 2021). RdRp was prepared as follows: The vector was transformed into *E*. *coli* BL21 (DE3) cells and cultured at 37 °C. When the optical density at 600 nm (OD_600_) reached 0.4–0.6, protein expression was induced by the addition of IPTG to a final concentration of 1 mM. The cells were harvested at 16 °C with shaking at 120 rpm for 20 h. Cells were harvested by centrifugation at 3,000 × g for 10 min at 4 °C and washed with cold D-PBS. The pellet was resuspended in xTractor Buffer (Takara) and homogenized by sonication (3 cycles of 30 s each). The lysate was centrifuged at 15,000 × g for 10 min at 4 °C to obtain the supernatant. The RdRp complex was purified using a His60 Ni Gravity Column Purification Kit (Takara) according to the manufacturer’s instructions.

The RNA-dependent RNA polymerase (RdRp) activity of SARS-CoV-2 was quantified by monitoring the synthesis of double-stranded RNA (dsRNA) from a single-stranded poly(C) RNA template (Sigma-Aldrich, Burlington, MA, USA) using fluorescence-based PicoGreen dye (Thermo Fisher Scientific, Waltham, MA, USA) (Giancotti et al., 2022). The 50-μL reaction mixture contained purified RdRp, 0.23 mM rGTP (Promega), 250 ng of poly(C) RNA, 2.5 mM MnCl_2_, 5 mM DTT, 0.01% bovine serum albumin, and 0.005% Tween 20, in 20 mM Tris-HCl buffer (pH 7.5). Samples or vehicle control (0.5% v/v DMSO) were pre-incubated with the RdRp reaction mixture (excluding rGTP) at room temperature for 10 min. The polymerization reaction was then initiated by the addition of rGTP and performed at 30 °C for 60 min in a Thermal Cycler Dice® Real-Time System (Takara). The reaction was terminated by adding 5 mM EDTA. Following termination, the reaction mixtures were transferred to black 96-well microplates, and 165 μL of PicoGreen solution (diluted 1:680 [v/v] in Tris and EDTA buffer, pH 7.5) was added. The fluorescence intensity was measured using a plate reader at excitation and emission wavelengths of 485 and 520 nm, respectively. For the RdRp assay, suramin was used as a positive control (Supplementary Figure S2) (Yin et al., 2021). The optimized gene encoding the RdRp of norovirus [GenBank accession No. AJ583672, nucleotides 987–2516] was cloned into the pET-28b (+) expression vector for recombinant protein expression in *E*. *coli*. Purification of norovirus RdRp and polymerase activity assays were performed using the procedures described above.

### 2.6 High-performance liquid chromatography and liquid chromatography–tandem mass spectrometry analyses and fractionation of *Ephedra sinica* samples (KES1–8)

Approximately 0.03 g of powdered *E*. *sinica* (KES1–8) was precisely weighed, and 1.5 mL of an extraction solvent, identical in composition to the mobile phase, was added. The mixture was then ultrasonically extracted at room temperature for 30 min. After sonication, the extract was centrifuged at 13,000 rpm for 10 min, and the resulting supernatant was filtered through a 0.45-μm membrane filter (Nippon Genetics, Tokyo, Japan) to obtain a clear solution for high-performance liquid chromatography (HPLC) and liquid chromatography–tandem mass spectrometry (LC-MS/MS) analyses. HPLC analysis was performed using a Hitachi L-2200 (Hitachi High-Technologies, Tokyo, Japan) system equipped with a COSMOSIL packed column 5C18-MS-II (4.6 mm I.D. × 150 mm). The mobile phase consisted of a mixture of CH_3_CN, H_2_O, H_3_PO_4_, and SDS in a ratio of 195 mL, 305 mL, 0.8 mL, and 2.4 g, respectively. Separation was performed at 40 °C with a flow rate of 1.0 mL/min. A 10-μL aliquot of the sample solution was injected, and detection was performed at 210 nm using a UV detector. Ephedrine (EP) and pseudoephedrine (PEP) were quantified using an external standard method with calibration curves prepared from EP hydrochloride and PEP hydrochloride standards. Total polyphenol content was determined using the Folin–Ciocalteu assay, as previously described (Kudo et al., 2023). The KES8 extract (20 mg) was applied to a column (2 × 40 cm) packed with silica gel in 5% MeOH/CHCl_3_. Elution was performed using a stepwise gradient of 5% (KES8 low-polarity fraction), 8%, 12%, 15%, 50%, and 75% MeOH/CHCl_3_ and MeOH (KES8 high-polarity fraction). The fractions were collected in 100-mL aliquots. The eluted fractions were evaporated and dissolved in mobile phase for LC-MS/MS analysis. LC-MS/MS analysis was performed using an LCMS-9030 (Shimadzu Corporation, Kyoto, Japan) equipped with an ESI source operated in positive ion mode. Chromatographic separation was achieved on a COSMOSIL 2.5C18-MS-II column (2.0 mm × 100 mm, 2.5 µm particle size) that was maintained at 40 °C. The mobile phases consisted of solvent A (water containing 0.1% formic acid) and solvent B (acetonitrile containing 0.1% formic acid). The gradient program was as follows: 98%–95% A (0–5 min), 95%–20% A (5–20 min), and 5% A (20–25 min). The flow rate was set at 0.5 mL/min. Mass spectra were acquired in full-scan mode over the m/z range of 50–1000. Metabolites of the high-polarity fraction were identified based on comparison with published reference spectra and previously reported compound assignments (Zang et al., 2013; Guo et al., 2024; Hu et al., 2024).

### 2.7 Statistical analysis

The SARS-CoV-2 infection assay was performed in two independent experiments, each with triplicate wells (n = 6). The other experiments were performed in three independent experiments (n = 3), unless otherwise stated. Data were analyzed using GraphPad Prism (version 10.0; GraphPad Software, San Diego, CA, USA). Statistical significance was evaluated using one-way analysis of variance, followed by Dunnett’s *post hoc* test. Statistical significance was set at *p* < 0.05. The screening data were visualized as a heatmap using the R software (version 4.5.1).

## 3 Results

### 3.1 Evaluation of SARS-CoV-2 infection in VeroE6/TMPRSS2 cells and S protein-mediated cell–cell fusion assay

**FIGURE 1:**
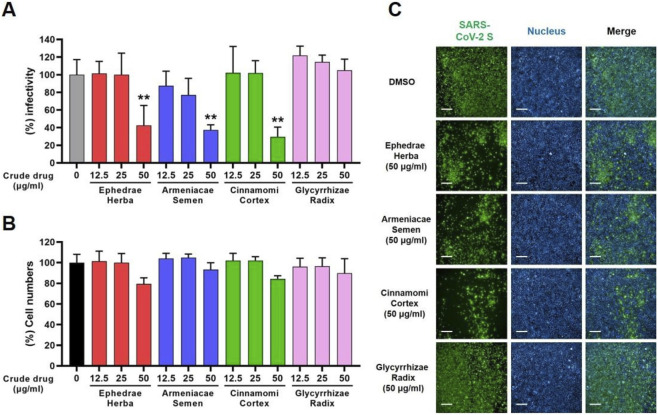
Inhibitory effects of the four crude drugs of Maoto on SARS-CoV-2 infection. **(A,B)** VeroE6/TMPRSS2 cells were infected with SARS-CoV-2 for 24 h in the presence of the indicated concentrations of the crude drug extracts constituting Maoto (Ephedrae Herba, Cinnamomi Cortex, Armeniacae Semen, and Glycyrrhizae Radix). The percentages of infectivity and cell numbers were normalized to the levels observed in cells treated with DMSO and infected with SARS-CoV-2. **(C)** Representative fluorescence images show the SARS-CoV-2 S protein in green and the cell nuclei in blue. Scale bar: 200 µm. Values represent the mean ± standard deviation of two independent experiments, each performed in triplicate wells (n = 6).

To evaluate the effects of Ephedrae Herba in comparison with the other crude drugs of Maoto (Armeniacae Semen, Cinnamomi Cortex, and Glycyrrhizae Radix), we performed SARS-CoV-2 infection assays (Figure 1). Glycyrrhizae Radix did not show a clear inhibitory effect on infection at any concentration tested. In contrast, at 50 μg/mL, Ephedrae Herba, Armeniacae Semen, and Cinnamomi Cortex reduced SARS-CoV-2 infection, with inhibition rates of 57.4%, 62.7%, and 70.4%, respectively. The corresponding relative cell numbers at 50 μg/mL were 79.4%, 93.3%, and 89.9%, respectively. Although slight reductions in cell number were observed, these reductions were not statistically significant.

**FIGURE 2:**
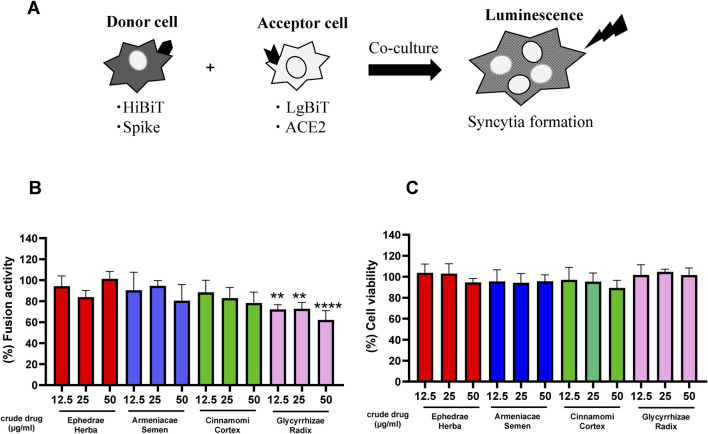
Inhibitory effects of the four crude drugs of Maoto on SARS-CoV-2 S protein-mediated cell–cell fusion. **(A)** Schematic model of the cell–cell fusion assay using a split-luciferase system. **(B)** Donor cells were mixed with acceptor cells in the presence of the test samples at the indicated concentrations (12.5, 25, and 50 μg/mL), and cell–cell fusion activity was evaluated. **(C)** Cell viability was measured after 24 h of treatment with each crude drug extract at the indicated concentrations (12.5, 25, and 50 μg/mL). Results are presented as the mean ± standard deviation of three independent experiments (n = 3).

Next, we evaluated inhibitory effects using an S protein-mediated cell–cell fusion assay as a model of syncytium formation (Figure 2) (Hörnich et al., 2021). Glycyrrhizae Radix significantly inhibited cell–cell fusion, with inhibition rates of 27.8%, 27.2%, and 39.2% at 12.5, 25, and 50 μg/mL, respectively. In contrast, Ephedrae Herba, Armeniacae Semen, and Cinnamomi Cortex did not significantly inhibit cell–cell fusion at the concentrations tested.

These comparative analyses showed that Ephedrae Herba, Armeniacae Semen, and Cinnamomi Cortex reduced SARS-CoV-2 infection in VeroE6/TMPRSS2 cells, whereas Glycyrrhizae Radix inhibited S protein-mediated cell–cell fusion.

### 3.2 Evaluation of protease inhibition by crude drugs

**FIGURE 3:**
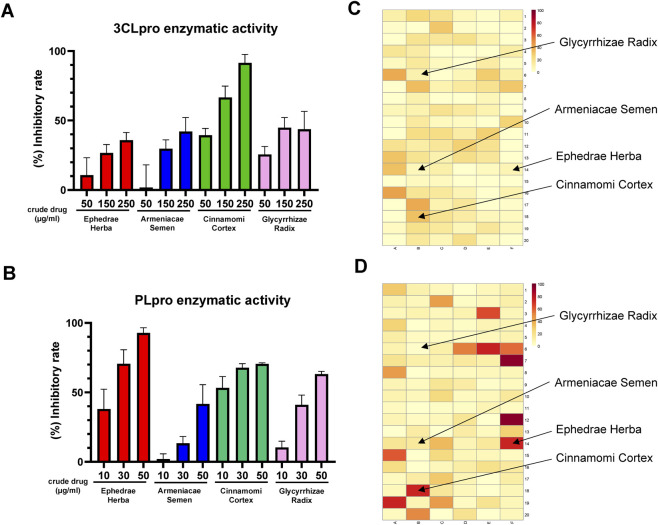
Inhibitory effects of the four crude drugs of Maoto on 3CLpro and PLpro activities. **(A,B)** 3CLpro and PLpro inhibitory effects of the four crude drug extracts at three concentrations (50, 150, 250 μg/mL for 3CLpro and 10, 30, 50 μg/mL for PLpro). Fluorescence intensity was measured using a fluorogenic substrate, and the inhibition rate was calculated. Data represent the mean ± standard deviation of three independent experiments (n = 3). **(C,D)** Heatmap data of screening of inhibitory activity against 3CLpro (100 μg/mL) and PLpro (20 μg/mL). In total, 120 crude drug extracts were used for the screening assays.

We next evaluated the inhibitory activities of the crude drugs against two key viral proteases, 3CLpro and PLpro, using *in vitro* enzymatic assays. Because different concentration ranges were used for the 3CLpro and PLpro assays, the four crude drugs of Maoto were compared using the intermediate concentration in each assay. Ephedrae Herba showed relatively lower inhibitory activity against 3CLpro, with an inhibition rate of 26.7%, whereas Cinnamomi Cortex, Armeniacae Semen, and Glycyrrhizae Radix showed inhibition rates of 66.6%, 29.8%, and 44.9%, respectively (Figure 3A). In contrast, Ephedrae Herba showed relatively high inhibitory activity against PLpro, with an inhibition rate of 70.6%, whereas Cinnamomi Cortex, Armeniacae Semen, and Glycyrrhizae Radix showed inhibition rates of 67.8%, 13.4%, and 41.0%, respectively (Figure 3B).

To place these activities in a broader comparative context, we screened 120 representative crude drugs commonly used in Kampo medicine and visualized the results as heat maps (Figures 3C,D). Cinnamomi Cortex showed moderate inhibitory activity against 3CLpro, ranking in the upper 40%–60%, and strong inhibitory activity against PLpro, ranking in the top 20%. Ephedrae Herba also ranked among the top 20% of crude drugs in the PLpro assay.

### 3.3 Evaluation of SARS-CoV-2 RdRp inhibition by Ephedrae Herba

**FIGURE 4:**
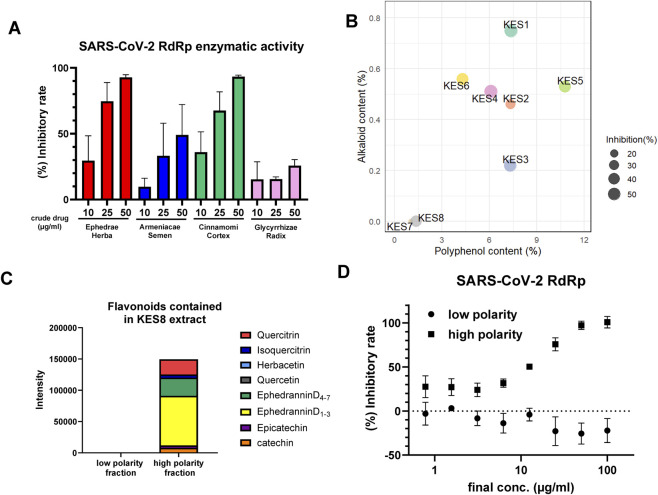
Inhibitory effects of Ephedrae Herba and related fractions on SARS-CoV-2 RdRp activity. RdRp inhibitory activity was evaluated using a fluorescence-based polymerase assay. **(A)** Each crude drug extract was tested at the indicated concentrations (10, 25, and 50 μg/mL). **(B)** Relationship between the phytochemical profiles of Ephedra sinica samples (KES1–8) and their inhibitory activity against SARS-CoV-2 RdRp. Dot size indicates the percentage of RdRp inhibition. **(C)** Flavonoid composition of the high- and low-polarity fractions of the KES8 extract analyzed using LC-MS/MS. **(D)** The high- and low-polarity fractions were evaluated for inhibitory activity against SARS-CoV-2 RdRp. Results are presented as the mean ± standard deviation of three independent experiments (n = 3).

Next, we evaluated the inhibitory effects of the four crude drugs of Maoto on SARS-CoV-2 RdRp (Figure 4A). Ephedrae Herba and Cinnamomi Cortex exhibited relatively high inhibitory activity, with inhibition rates of 74.6% and 67.5%, respectively, at a concentration of 25 μg/mL. To further examine the metabolites associated with the RdRp inhibitory activity of Ephedrae Herba, we analyzed eight cultivated *E. sinica* strains (KES1–8), the botanical source of Ephedrae Herba, which exhibited different phytochemical profiles (Figure 4B). Because Ephedrae Herba contains alkaloids such as ephedrine as well as polyphenolic metabolites, we assessed the relationship between RdRp inhibitory activity and the contents of alkaloids and total polyphenols in these samples. Among the eight samples, KES1–6 contained moderate to high levels of alkaloids and polyphenols, whereas KES7 and 8 contained low levels of both. All extracts except KES7 showed moderate inhibitory activity (Figure 4B). Correlation analysis revealed positive associations of RdRp inhibitory activity with total polyphenol and alkaloid contents (Spearman’s r = 0.38 and 0.41, respectively), although neither correlation reached statistical significance (p > 0.05; Supplementary Table S1). These results suggest that inhibitory activity cannot be explained simply by the total amounts of alkaloids or polyphenols. Notably, among the samples lacking detectable alkaloids and containing low levels of polyphenols, KES7 showed weak inhibitory activity (13.3% ± 3.3%), whereas KES8 retained moderate inhibitory activity (41.4% ± 1.8%).

Therefore, we further fractionated KES8 into high- and low-polarity fractions and compared their inhibitory activities. The LC–MS/MS analysis revealed enrichment of the high-polarity fraction with flavonoids, including ephedrannin-D_1-3_, ephedrannin-D_4-7_, quercitrin, quercetin, isoquercitrin, herbacetin, epicatechin, and catechin (Figure 4C). The high-polarity fraction exhibited concentration-dependent inhibition of RdRp, with an IC_50_ of 8.6 μg/mL, whereas the low-polarity fraction showed no such effect (Figure 4D).

**FIGURE 5:**
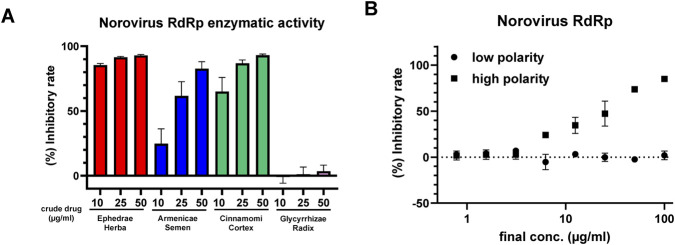
Inhibitory effects of Ephedrae Herba and related fractions on norovirus RdRp. **(A)** Each extract was tested at the indicated concentrations (10, 25, and 50 μg/mL). **(B)** The high- and low-polarity fractions of the KES8 extract were evaluated for inhibitory activity against norovirus RdRp. Results are presented as the mean ± standard deviation of three independent experiments (n = 3).

To determine whether similar inhibition occurs against the RdRp of other viruses, we evaluated the inhibitory effects of the four crude drugs of Maoto on norovirus RdRp. We previously reported inhibitory effects of Ephedrae Herba extract on human norovirus replication in human intestinal organoids (Hayashi et al., 2023). The Ephedrae Herba extract also demonstrated inhibitory activity against norovirus RdRp, and its high-polarity fraction exhibited concentration-dependent inhibition, with an IC_50_ of 16.5 μg/mL (Figures 5A,B).

## 4 Discussion

In the present study, we focused on Ephedrae Herba and comparatively examined the four crude drugs of Maoto—Ephedrae Herba, Armeniacae Semen, Cinnamomi Cortex, and Glycyrrhizae Radix—using a SARS-CoV-2 infection assay, an S protein-mediated cell–cell fusion assay, and *in vitro* assays targeting virus enzymes (3CLpro, PLpro, and RdRp). We found that each crude drug exhibited a distinct inhibitory profile across the different assay systems.

In the infection assay, Ephedrae Herba, Armeniacae Semen, and Cinnamomi Cortex reduced SARS-CoV-2 infection, whereas Glycyrrhizae Radix did not exhibit a clear inhibitory effect. This pattern was generally consistent with a previous report in which Ephedrae Herba showed antiviral activity, while the effect of Glycyrrhizae Radix was limited (Kakimoto et al., 2022). In contrast, in the cell–cell fusion assay used in the present study, Glycyrrhizae Radix inhibited S protein-mediated cell–cell fusion. These findings suggest that Glycyrrhizae Radix may affect fusion-related processes rather than suppressing infection in the same manner as the other crude drugs. In addition, glycyrrhizic acid, a major metabolite of Glycyrrhizae Radix, has been reported to interfere with processes related to the interaction between ACE2 and the SARS-CoV-2 S protein (Yu et al., 2021), which may be relevant to the fusion-inhibitory effect observed in the present study.

With respect to protease inhibition, Cinnamomi Cortex exhibited relatively high inhibitory activity against 3CLpro, whereas Ephedrae Herba and Cinnamomi Cortex also exhibited relatively high inhibitory activity against PLpro. Previous studies reported that cinnamtannin B2, a metabolite of Cinnamomi Cortex, may inhibit SARS-CoV-2 3CLpro *in silico* (Rajendran et al., 2022). In addition, we previously showed that tea-derived polyphenol fractions inhibit PLpro (Kiba et al., 2024). Because Ephedrae Herba and Cinnamomi Cortex are rich in polyphenols (Shi et al., 2023; Tang et al., 2023), some of these metabolites may be involved in the inhibitory activities observed here. However, we here demonstrated the inhibitory effects of these crude drugs on 3CLpro and PLpro *in vitro* assays, and the extent to which they contribute to the antiviral effects observed in cell-based assays remains to be determined. Because GST-tagged 3CLpro was used in the present assay owing to substantial loss of activity after tag removal, a possible influence of the N-terminal fusion tag on enzymatic behavior should be considered when interpreting the 3CLpro inhibition data.

Another important finding of this study is that Ephedrae Herba and Cinnamomi Cortex exhibited relatively high inhibitory activity against SARS-CoV-2 RdRp. We focused on Ephedrae Herba and performed comparative analyses of different Ephedra sinica samples (KES1–8) as well as fractionation studies. These results indicated that the observed RdRp inhibitory activity was not fully explained by alkaloid or total polyphenol contents alone, since activity was also retained in the ephedrine-free high-polarity fraction. This fraction was enriched in flavonoids and exhibited concentration-dependent inhibitory effects against both SARS-CoV-2 RdRp and norovirus RdRp. A recent study reported that theaflavin-3,3'-digallate, a natural tea-derived polyphenol-related product, inhibits RdRp (Li C. W. et al., 2024); the activity observed in the present high-polarity fraction may be consistent with these findings. However, in this study we did not identify a single active metabolite or the underlying mode of action, and metabolites in the high-polarity fraction may have contributed to the observed activity.

Several limitations of this study should be acknowledged. RdRp inhibitory activity was demonstrated only in biochemical enzyme assays. It can thus not directly explain the antiviral effects observed in the cell-based infection assay. In addition, although cell number was evaluated in parallel with infectivity by counting DAPI-positive nuclei, a dedicated cytotoxicity assay using sample treatment alone was not independently performed. Quantitative pharmacological indices such as EC_50_, CC_50_, and the selectivity index were not determined. Moreover, while the infection assay was based on a previously reported imaging-based method and infectivity was assessed relative to the vehicle-treated infected control, a dedicated positive control was not included in the present study. Furthermore, the VeroE6/TMPRSS2 system is a useful screening model but does not fully reproduce the physiology of the human respiratory epithelium. Finally, although RdRp-related inhibitory activity was enhanced in the high-polarity fraction of Ephedrae Herba, the underlying active metabolite(s) and their mode of action remain unclear. These issues should be addressed in future studies using more physiologically relevant respiratory models and further chemical and pharmacological characterization.

The present study comparatively evaluated the effects of the four crude drugs of Maoto on SARS-CoV-2 infection, S protein-mediated cell–cell fusion, and virus enzymes, with a particular focus on Ephedrae Herba. Ephedrae Herba and Cinnamomi Cortex showed relatively high inhibitory activity against RdRp, and the flavonoid-rich high-polarity fraction of Ephedrae Herba exhibited concentration-dependent inhibitory effects against both SARS-CoV-2 RdRp and norovirus RdRp. These findings suggest that high-polarity metabolites of Ephedrae Herba may contribute to RdRp-related inhibitory activity. However, because the present study was based mainly on *in vitro* and biochemical assays, the findings provide only limited insight into the overall antiviral effects of Ephedrae Herba. Further studies using physiologically more relevant models and identification of active metabolites are necessary.

## Data Availability

The original contributions presented in the study are included in the article/Supplementary Material, further inquiries can be directed to the corresponding authors.
