# Supplementary material for: Ephedrae Herba as a potential source of SARS-CoV-2 RNA-dependent RNA polymerase inhibitory activity
Source: Front Pharmacol. 2026 Jul 2;17:1718569. doi: 10.3389/fphar.2026.1718569 (PMC13372621; doi:10.3389/fphar.2026.1718569)
Supplement: Supplementary file 1 [file Presentation1.pdf]

Supplementary Figure S1

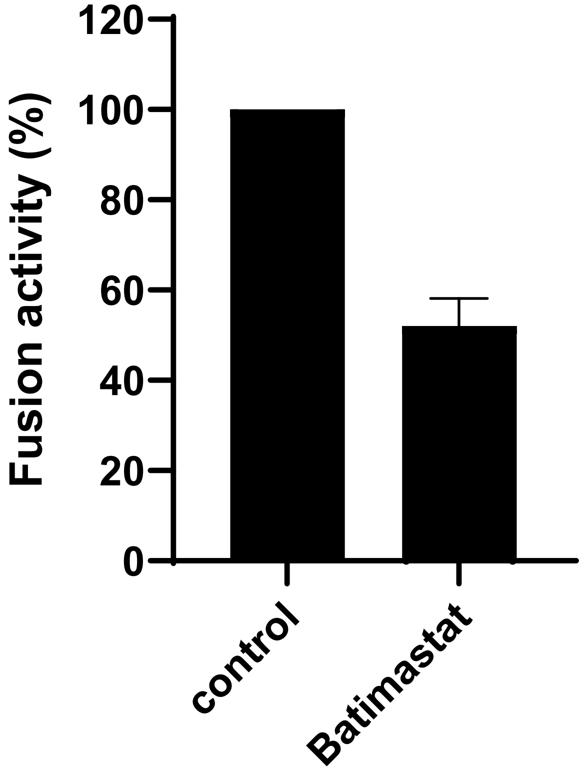

Fig. S1 SARS-CoV-2 S protein mediated cell-cell fusion assay. Batimastat was used as a positive control and inhibited S protein-mediated cell-cell fusion. Data are presented as mean ± SD from independent experiments (n = 4).

Supplementary Figure S2

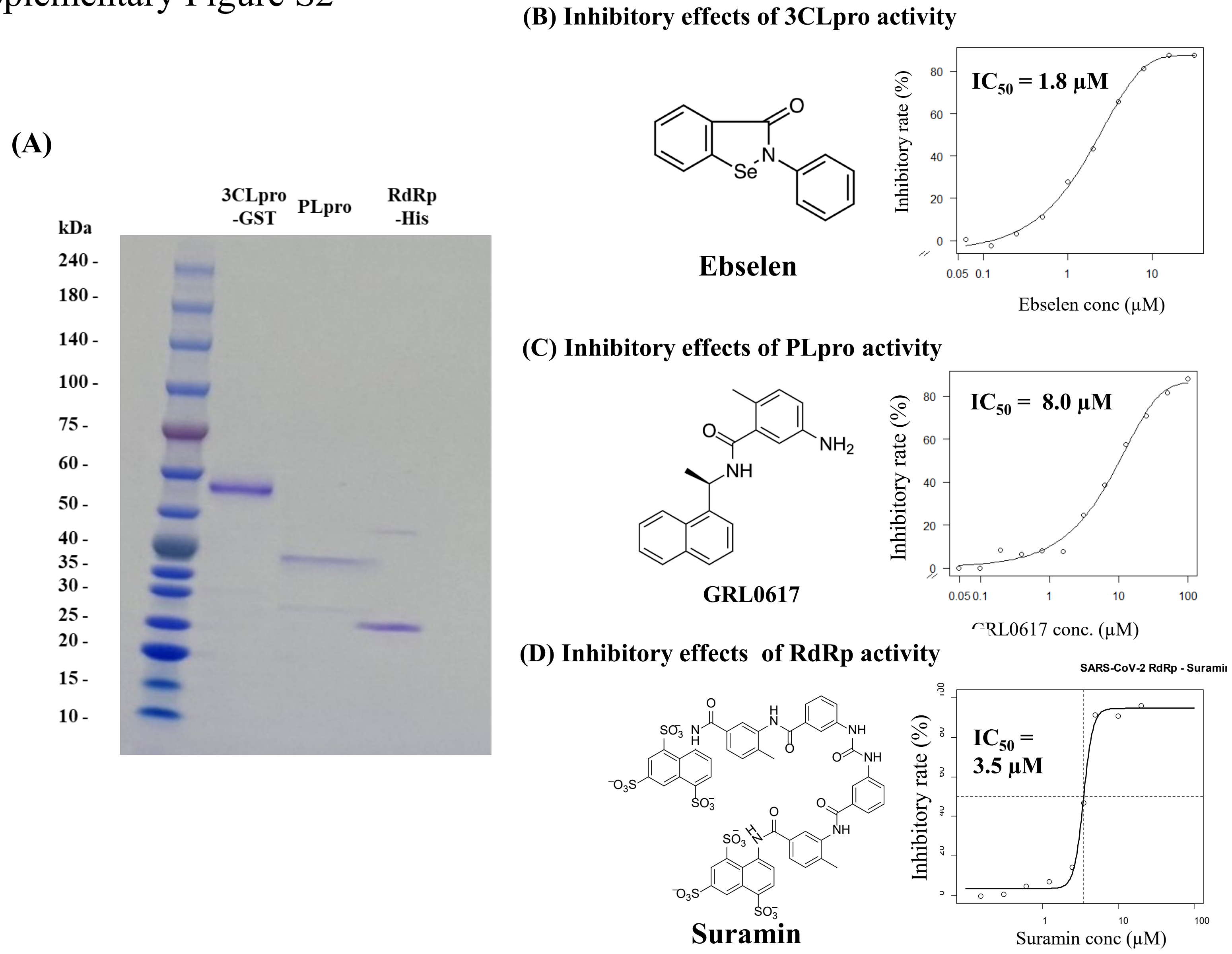

Fig. S2 Fluorogenic in vitro activity assays for SARS-CoV-2 3CLpro, PLpro, and RdRp. (A) Purified GST-3CLpro, PLpro, and RdRp-His were separated by 12% SDS-PAGE and stained with CBB. (B) The 3CLpro activity assay was validated using the 3CLpro inhibitor ebselen. (C) The PLpro activity assay was validated using the PLpro inhibitor GRL0617. (D) The RdRp activity assay was validated using the RdRp inhibitor suramin.

Supplementary Table S1 SARS-CoV-2 RdRp inhibitory activities and phytochemical contents of 8 *Ephedra sinica* samples (KES1-KES8)

Sample ID	Inhibitory rate (%)	Total polyphenol (%)	Alkaloids (%)					
			Total alkaloid	NPE*1	NE*1	PE*1	E*1	ME*1
KES1	57.6 ± 1.9 %	7.36	0.75	0.03	0.05	0.23	0.52	0.02
KES2	28.6 ± 3.4 %	7.34	0.46	0.04	0	0.18	0.28	0.02
KES3	50.2 ± 2.4 %	7.32	0.22	0.01	0	0.16	0.06	0.01
KES4	57.4 ± 0.8 %	6.09	0.51	0.01	0.1	0.05	0.45	0.02
KES5	44.0 ± 3.4 %	10.76	0.53	0.01	0.06	0.21	0.32	0.02
KES6	42.6 ± 2.0 %	4.29	0.56	0.01	0	0.19	0.37	0.05
KES7	13.3 ± 3.3 %	1.03	0	0	0	0	0	0
KES8	41.4 ± 1.8 %	1.36	0	0	0	0	0	0
Correlation coefficient*2		0.38	0.41					

*1 Norpseudoephedrine (NPE), norephedrine (NE), pseudoephedrine (PE), ephedrine (E), and methylephedrine (ME). *2 Spearman's r correlation coefficients between RdRp inhibition and polyphenol or alkaloid levels.
